# Polygenic risk associated with Alzheimer’s disease and other traits influences genes involved in T cell signaling and activation

**DOI:** 10.3389/fimmu.2024.1337831

**Published:** 2024-03-25

**Authors:** Dallin Dressman, Shinya Tasaki, Lei Yu, Julie Schneider, David A. Bennett, Wassim Elyaman, Badri Vardarajan

**Affiliations:** ^1^ Department of Neurology, Columbia University, New York, NY, United States; ^2^ The Taub Institute for Research on Alzheimer’s Disease and the Aging Brain, Columbia University, New York, NY, United States; ^3^ Rush University Medical Center, Rush Alzheimer’s Disease Center, Chicago, IL, United States; ^4^ Department of Neurological Sciences, Rush University Medical Center, Chicago, IL, United States; ^5^ Department of Pathology, Rush University Medical Center, Chicago, IL, United States; ^6^ College of Physicians and Surgeons, Columbia University, The New York Presbyterian Hospital, The Gertrude H. Sergievsky Center, New York, NY, United States

**Keywords:** polygenic risk score, Alzheimer’s disease, T cells, gene expression, genotype phenotype correlation, immunology

## Abstract

**Introduction:**

T cells, known for their ability to respond to an enormous variety of pathogens and other insults, are increasingly recognized as important mediators of pathology in neurodegeneration and other diseases. T cell gene expression phenotypes can be regulated by disease-associated genetic variants. Many complex diseases are better represented by polygenic risk than by individual variants.

**Methods:**

We first compute a polygenic risk score (PRS) for Alzheimer’s disease (AD) using genomic sequencing data from a cohort of Alzheimer’s disease (AD) patients and age-matched controls, and validate the AD PRS against clinical metrics in our cohort. We then calculate the PRS for several autoimmune disease, neurological disorder, and immune function traits, and correlate these PRSs with T cell gene expression data from our cohort. We compare PRS-associated genes across traits and four T cell subtypes.

**Results:**

Several genes and biological pathways associated with the PRS for these traits relate to key T cell functions. The PRS-associated gene signature generally correlates positively for traits within a particular category (autoimmune disease, neurological disease, immune function) with the exception of stroke. The trait-associated gene expression signature for autoimmune disease traits was polarized towards CD4+ T cell subtypes.

**Discussion:**

Our findings show that polygenic risk for complex disease and immune function traits can have varying effects on T cell gene expression trends. Several PRS-associated genes are potential candidates for therapeutic modulation in T cells, and could be tested in *in vitro* applications using cells from patients bearing high or low polygenic risk for AD or other conditions.

## 1 Introduction

Alzheimer’s disease (AD) is a chronic neurodegenerative condition that afflicts millions of Americans. While AD pathology has long been known to include aberrant aggregation of amyloid beta peptide and tau protein, it is also linked to inflammation and other immune processes. T cells comprise part of the adaptive immune response to pathogens and other biological insults. Recently, T cells in AD patients have shown a high degree of clonal expansion, a less diverse T cell receptor repertoire, increased infiltration into the cerebrospinal fluid (CSF) and brain parenchyma, and upregulation of genes involved in cytotoxicity, inflammation, immunosenescence, and response to certain chemokines (1–3). Researchers have previously detailed how T cell gene expression changes are correlated with individual genetic variants, some of which are associated with AD and other diseases (4–11). Several of these studies also profile T cells at various stages of differentiation or activation using flow-assisted cell sorting or single-cell RNA-sequencing, and show that many genotype-dependent gene expression changes are specific to particular T cell subtypes or activation states. However, aggregating the effects of many genetic variants may better capture genotype-phenotype correlation in complex disease, especially if these variants have been previously linked to disease risk. Thus, we now focus on correlating polygenic risk scores (PRSs) for AD and other conditions with T cell gene expression data, building on research using PRSs to better understand AD and related phenotypes.

While novel approaches to PRS studies are accelerating, few studies have correlated PRSs with gene expression at this stage (12–14), and no studies, to our knowledge, have correlated a PRS for any disease with T cell gene expression. Because our patient cohort consists of AD patients and age-matched healthy controls from the Religious Orders Study and Memory and Aging Project (15), we first calculate a PRS for AD, validating it against diagnostic data and neuropathological measurements. We then correlate gene expression data from four T cell subtypes with the PRS for AD, and with PRSs for 13 other immune cell, autoimmune disease, and neurological disease traits. We differentiated these T cell subtypes between CD4+ *vs*. CD8+ and naïve *vs*. memory populations, to better capture the reality of cell type-specific or state-specific effects of genetic variants on gene expression that has been shown in other studies (6, 8–11).

We aim to understand the differential gene expression in T cells at different polygenic risk levels for AD and other disorders. We hypothesize that disease-relevant genes and pathways will be differentially expressed with respect to polygenic risk for disease. We expect that our dataset, involving four T cell subtypes, will highlight differences in PRS-associated genes across T cell subtypes and disease traits. Our findings highlight biological pathways and other mechanisms of polygenic risk for disease, which can aid in hypothesis generation for future targeted studies of T cell behavior in AD and other conditions. They also provide interesting comparisons to previous genotype-phenotype correlation studies using T cell RNA-sequencing data (4–11).

## 2 Materials and methods

### 2.1 Study participants

Study participants come from the Religious Orders Study (ROS) and Memory and Aging Project (MAP), described in detail elsewhere (15). Briefly, ROS enrolls Catholic priests, nuns, and brothers, without known dementia, aged 53 or older from more than 40 groups in 15 states across the USA. MAP enrolls men and women without known dementia aged 55 or older from northeastern Illinois. Peripheral blood mononuclear cells (PBMC) from 96 ROSMAP participants were used in this study. 48 participants were clinically and/or pathologically diagnosed with AD, while 48 participants without dementia served as controls. Brain tissue from each participant was analyzed post-mortem to detect pathological signs of neuritic plaques and neurofibrillary tangles.

### 2.2 Sample preparation, RNA-sequencing, and genotyping

PBMCs were isolated by Ficoll gradient centrifugation, then sorted by high-speed flow cytometry into the following T cell subtypes: CD4+CD45RO-, CD4+CD45RO+, CD8+CD45RO-, and CD8+CD45RO+. Because CD4+ T cell subtypes such as Th1, Th2, Th17, and regulatory T cells are best distinguished by intracellular markers that cannot be detected prior to fixation and permeabilization, and to ensure sufficient post-sorting cell counts for RNA extraction, we chose to forgo isolation of CD4+ T cell subtypes beyond the presence or absence of CD45RO. Total RNA was extracted using buffer TCL (Qiagen), then RNA-seq libraries were prepared according to the Single Cell RNA Barcoding and Sequencing method originally developed for single-cell RNA-seq (16), adapted for extracted total RNA. RNA libraries were collected on a single 384-well plate and sequenced on the Illumina HiSeq using the High-throughput 3’ Digital Gene Expression (DGE) library (16). Genes with maximum count value of at least three and non-zero values in over twenty percent of samples were included in differential expression analysis. Expression values were normalized to counts per million (CPM). The EdgeR package in R (17) was used to conduct differential expression, and Voom transformation (18) was applied to gene expression data. DNA for genotyping was extracted from whole blood or frozen post-mortem brain tissue and genotyped using the Affymetrix GeneChip 6.0 platform. Quality control of genotyping data was done with PLINK (19) (http://pngu.mgh.harvard.edu/~purcell/plink/), and imputation was done with MACH software (version 1.0.16a).

### 2.3 Polygenic risk score calculation

Summary statistics files from genome-wide association studies were used as the base data. Duplicate SNPs were removed from base data files, as were ambiguous SNPs for which the effect allele and other allele were complementary nucleotides (C with G or A with T), using PLINK (19). For summary statistics whose coordinates were found on genome build 38, LiftOver (http://genome.ucsc.edu) was used to convert these coordinates to genome build 37, to match the target data. For traits with missing odds ratio values in the summary statistics, these were calculated from the beta values by using the exp() function in R. For traits with missing beta values, beta values were estimated using the sample size, Z-score, and allele frequencies. The estimated beta values were then converted to odds ratio values using exp().

Genomic data from ROSMAP participants was used as the target data. These data were stored as three separate batches (ROSMAP_n1686, ROSMAP_n381, and ROSMAP_BU) due to separate genotyping batches, and initially processed individually by cohort. Quality control of target data was done with R and PLINK (19). First, SNPs were filtered to exclude those with a minor allele frequency (MAF) less than 0.01, a Hardy-Weinberg Equilibrium test *p*-value under 1 x 10^-6^, and SNPs missing in at least 1% of participants. Individuals missing over 1% of SNPs in their genotyping data were also excluded at this stage. We then pruned highly correlated SNPs using a window size of 200 variants, a step size of 50 variants at a time, and filtered out any SNPs with an LD *r* ^2^ value above 0.25. Participants with heterozygosity F coefficients greater than three standard deviations from the mean, with differences between reported sex and sex chromosomes, or with a first or second degree relative in the sample, were excluded.

After these quality control steps on individual batches, we used PLINK to merge ROSMAP_n1686, ROSMAP_n381, and 15 participants from ROSMAP_BU. We limited the inclusion of participants from ROSMAP_BU to participants with T cell gene expression data whose AD PRS was not an outlier in the overall distribution. This was because of the high number of unique SNPs in the ROSMAP_BU cohort relative to ROSMAP_n1686 and ROSMAP_n381, which we suspect is due to low-quality imputation of rare variants. In the merged dataset, we excluded SNPs with MAF < 0.01 and SNPs missing in at least 5% of participants. The merged dataset was then used as target data in further PRS calculation.

We used PRSice-2 (20) for PRS calculation. PRSice-2 uses the standard C+T approach, meaning that PRSice-2 first performs clumping of input variants using parameters of a 250 kb window, *p*-value threshold of 1, and *r*2 value threshold of 0.1. The user also inputs one or more *p-*value thresholds, such that the software will only include SNPs with a *p-*value below the threshold in PRS calculation. The software also automatically performs strand-flipping for SNPs whose alleles mismatch between base and target data. The software then adds the effects of individual SNPs as weighted by odds ratio for PRS calculation. We used age and sex as covariates. For the AD PRS, clinical AD diagnosis was used as the input phenotype, and SNPs within 1 Mb of the APOE locus were excluded as input in PRS calculation. Including genotyping batch as an additional covariate did not change the calculated PRSs. We input a range of *p*-value thresholds for SNP inclusion from 5 x 10^-8^ to 1, yielding a set of PRS scores as output for each individual.

### 2.4 Validation of AD PRS against clinical and pathological data

The pROC package (21) was used to calculate receiver operator characteristic (ROC) curves for the AD PRS against clinical AD diagnosis and against pathological AD diagnosis, at each SNP *p*-value threshold. We also used this package to calculate the predictive value of the AD PRS from the area under the ROC curves, to determine which *p*-value threshold yielded the highest predictive value. We compared the distribution of AD PRS scores at this *p*-value threshold between AD and non-AD participants using student’s T test. We also ran comparisons of the AD PRS at this *p*-value threshold against Braak score using nonparametric one-way ANOVA, and quantitative pathological measurements (amyloid plaque burden, tau tangle burden, and global pathology measurement) using linear regression with age, sex, clinical AD status, pathological AD status, and the first ten principal components from genotyping data as covariates.

### 2.5 Detection and pathway analysis of PRS-associated genes

Genotyping data and T cell RNA-sequencing data were available for 78 participants, with one participant excluded whose AD PRS was an outlier. For each trait and each T cell subtype, we ran linear regression of gene expression counts against PRS scores to detect PRS-associated genes using the lm() function in R. For covariates, we used age, sex, AD diagnosis (clinical and pathological), and the first 10 principal components from genotyping, with no interactions between covariates. Genes expressed in fewer than 20% of participants, or with a maximum expression count under 3, were excluded from PRS association analyses. Because non-AD traits did not have relevant phenotypic data for PRS validation, we used the same *p*-value threshold (p = 1) as the optimum PRS for all traits. For pathway analysis of PRS-associated genes, we used Gene Set Enrichment Analysis (GSEA (22)) separately for each T cell subtype and each trait. GSEA was run using the t-value from the PRS-gene association as the ranking metric, using the default value of 1,000 permutations and restricting the gene sets to those with 15-200 genes, using the GO Biological Processes 2022 database (23). We then detected biological pathways which were significantly over-represented or under-represented at a significant level of 0.05, after correcting for false discovery rate. Figures were generated using the ComplexHeatmap (24), cowplot, and ggplot2 (25) packages in R.

Genes associated with the PRS for AD were compared to a published single-cell RNA-sequencing dataset of peripheral blood from AD patients and healthy controls (26). We downloaded this dataset from the Gene Expression Omnibus (accession number GSE181279) and processed the data using the Seurat package in R (27). We first conducted a standard quality control and data normalization and transformation workflow for each individual in the dataset, then integrated the Seurat objects using the top 5000 highly variable genes as integration features. In the integrated object, we removed cells with over 15% mitochondrial genes, cells containing under 200 or over 3000 genes, and cells containing over 10,000 unique molecular identifiers, leaving 36,209 cells. We then reran quality control, normalization, transformation, and dimensionality reduction with principal component analysis, and clustered the cells with a resolution of 0.8. We identified clusters with high expression of CD3E and re-clustered them as T cells, rerunning the same quality control steps as in the integrated object, leaving 26,515 T cells. We then examined expression of canonical T cell subtype markers in each cluster to define clusters of CD4+ naïve, CD4+ memory, CD8+ naïve, and CD8+ memory T cells, corresponding to the four T cell subtypes in our PRS dataset. We used the FindMarkers function to detect differentially expressed genes between the AD subjects and healthy controls for the four T cell subtypes.

## 3 Results

### 3.1 Calculation and validation of a polygenic risk score for AD

We first used PRSice-2 (20) to calculate a genome-wide polygenic risk score for AD, for 2051 individuals in ROSMAP. We used the summary statistics from the Kunkle et al., 2019 (28) genome-wide association study (GWAS) for SNP effect sizes excluding SNPs in the APOE locus (see Methods). PRSs were calculated using SNPs associated with AD at *p*-value thresholds ranging from 5 x 10^-8^ to 1 (all SNPs), and PRS distributions at each threshold were standardized to have a mean of 0 and standard deviation of 1. For individuals with clinical or pathological AD diagnostic data, we computed the predictive value of the AD PRS for each *p*-value threshold (see **Supplementary Figure 1**). Using SNPs at a *p*-value threshold of 0.75 or 1 resulted in the highest predictive value of the PRS. We also found that the AD PRS correlated significantly with other measures of AD neuropathology, including Braak staging of tau pathology, amyloid burden, tau tangle burden, and global pathology.

### 3.2 Correlation of gene expression with PRSs for AD and other traits

Prior to correlating PRS with gene expression, we hypothesized that individuals in our cohort could possess genetic risk variants for other neurodegenerative disorders as well. Because other neurological disorders, such as epilepsy and stroke, involve acute damage to the blood-brain barrier with potential infiltration of peripheral immune cells into the CNS (29, 30), polygenic risk for these conditions could be linked to T cell-mediated neuroinflammation. We also hypothesized that T cell gene expression trends could be affected by polygenic risk for traits related to immune function or autoimmune disease, even without clinical presentation of these conditions in our cohort. Thus, we computed PRSs for common traits or diseases in these categories, including lymphocyte counts (31), white blood cell counts (31), C-reactive protein levels (31), ulcerative colitis (32), Crohn’s disease (32), multiple sclerosis (33), rheumatoid arthritis (34), systemic lupus erythematosus (35), type 1 diabetes (36), Parkinson’s disease (37), amyotrophic lateral sclerosis (38), epilepsy (39), and stroke (40). The GWAS studies used as base data in PRS calculation did not distinguish between phenotypic subtypes in disease cases, with the exception of rheumatoid arthritis, which additionally analyzed variants in seropositive patients after the case-control comparison.

78 participants with PRSs calculated from genotyping array data had T cell RNA-sequencing data from blood samples. One of these participants was excluded because their AD PRS was an outlier at the PRS distribution for *p* = 1, leaving 77 participants for correlation between PRSs and gene expression. Demographics for these participants, including AD diagnostic information, are given in **Table 1**. Each participant had bulk RNA-sequencing data from the following T cell subtypes, sorted using flow cytometry: CD4+CD45RO-, CD4+CD45RO+, CD8+CD45RO-, and CD8+CD45RO+. The CD45RO marker was used to separate putatively naïve from memory T cell populations to distinguish PRS-associated genes by T cell differentiation state, although a subset of memory T cells are now known to lose CD45RO expression and re-express CD45RA (41). In these participants, we computed the PRSs using all independent SNPs in the genome (*p*-value threshold of 1), since we did not have phenotypic data for PRS optimization, and the optimal PRS for AD was derived from the higher *p*-value thresholds. We then correlated the PRSs with T cell gene expression (see Methods for quality control measures used at this stage). 46 of our sequencing participants also have brain RNA-sequencing data from a recent publication (13), although we focus only on T cell gene expression here. A listing of all PRS-associated genes by trait and T cell subtypes is found in **Supplementary Table 1**.

**Table 1:** Summary of demographics for patients with data used for PRS-associated gene calculation.

Number of genotyped subjects with RNA-sequencing	77
Mean age at blood draw ± SD	79.9 ± 6.06
Women	54 (70.1%)
Number of autopsies	51 (66.2%)
Number of AD diagnoses based on pathology	30 (39.0%)
Number of AD diagnoses based on clinical signs	29 (37.7%)
Number of both clinical and pathological AD diagnoses	16 (20.8%)

**Figure 1:**
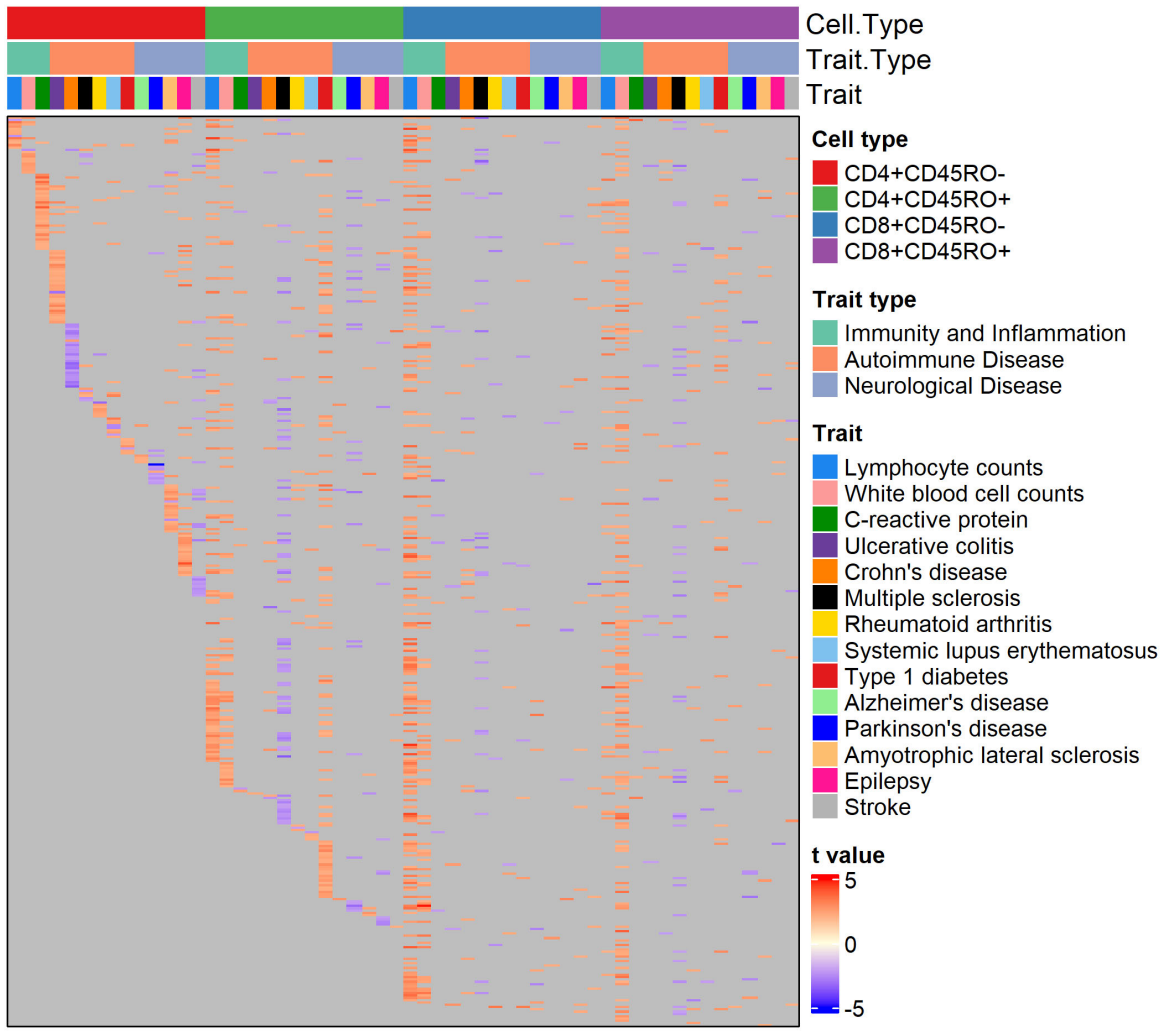
Heatmap of genes nominally associated with the PRS for one or more traits. Annotation rows above the heatmap show cell type, trait type, and trait, matching the color legends at the right. Each row of the heatmap is a gene, and each cell is colored by the strength and direction of the association with the PRS (shown by the color legend at bottom right), or gray if the association is insignificant.

Genes with a nominally significant relationship with the PRS are shown on the heatmap in **Figure 1**. Overall, 5961 genes (of 6139 genes passing minimum expression thresholds) were associated with the PRS for at least one trait in at least one cell type. We compared genes associated with the PRS for AD to a published single-cell RNA-sequencing dataset of peripheral blood mononuclear cells in AD patients and healthy controls (26). After identifying cell clusters from the single-cell data analogous to the four T cell subtypes in our dataset (see **Supplementary Figure 2A**), we found genes in these clusters with differential expression in AD patients versus controls. Of the genes found in both datasets that were significantly associated with the AD PRS in our cohort, 4-33% of them were upregulated with high AD PRS in our dataset and with AD status in the single-cell dataset, depending on the T cell subtype. These genes include MAPK1 in CD8+CD45RO+, which represses interferon signaling and is a key mediator of signaling pathways that promote cell proliferation and differentiation (42), C1QBP in CD4+CD45RO+, which promotes T cell survival and proliferation (43), and several genes involved in downstream signaling of the T cell receptor, such as DBNL in CD4+CD45RO-, NFATC2 in CD4+CD45RO+, and ITSN2 in CD8+CD45RO+ ( (44–46), see **Supplementary Figures 2B-F**). For a full listing of genes differentially expressed in AD patients for each of the four T cell subtypes in the comparison dataset, see **Supplementary Table 2**).

About 50% of PRS-associated genes were significantly associated with the PRS in four or more trait/cell type combinations (see **Supplementary Figure 3** for breakdown of trait overlap across and within cell types). Interestingly, several autoimmune disease traits feature almost all significant PRS-associated genes in an inverse relationship with the PRS (as seen by cells colored blue), regardless of the cell type, suggesting a pattern of downregulated genes in individuals with high polygenic risk for these conditions. 364 genes were nominally associated with the PRS for over ten traits across T cell subtypes. Among them were genes that play a role in T cell memory and activation, T cell receptor signaling, and cytokine response. These include TRAT1, SLA, IL10RA, MAF, and CXCR4. CXCR4 has potential disease relevance as a chemokine receptor that could mediate infiltration of T cells into the central nervous system (CNS) (47, 48). Interestingly, SOD1, a gene with risk variants for ALS (49), was associated with the PRS for several non-ALS traits in our cohort.

**Figure 2:**
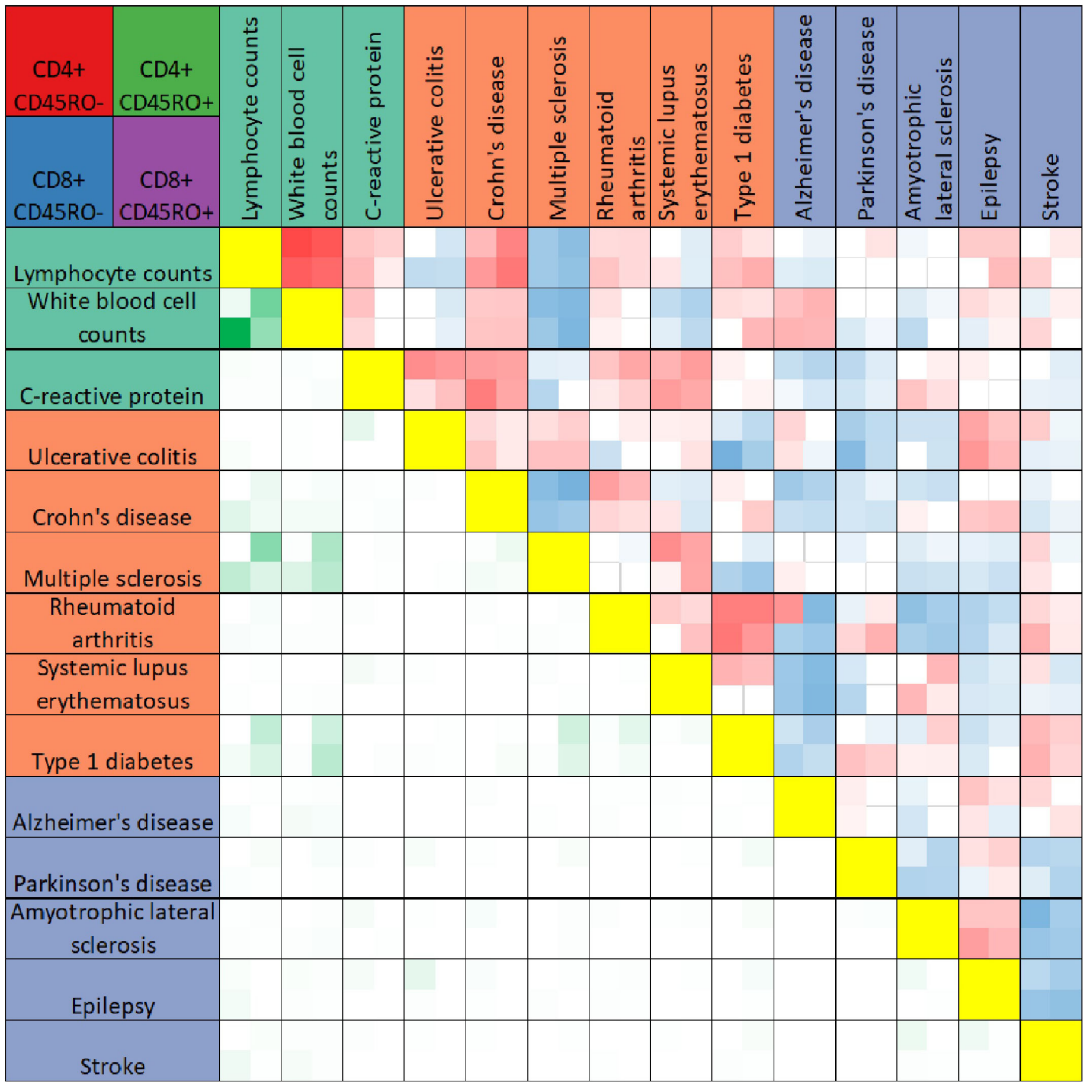
Heatmap comparison of PRS-associated genes across traits. The heatmap summarizes results of Pearson’s correlation test between PRS-associated genes for any two traits (boxes above yellow diagonal) or the numbers of genes significantly associated with the PRS (*p* < 0.05) shared between any two traits (boxes below the yellow diagonal). Traits are listed above and to the left of the heatmap, colored according to trait categories as in Figure 2. Each box in the heatmap reflects four values, for CD4+CD45RO- (top left), CD4+CD45RO+ (top right), CD8+CD45RO- (bottom left) and CD8+CD45RO+ (bottom right). Boxes for Pearson’s correlation tests are darker red for r values approaching 1, darker blue for r values approaching -1, and white if insignificant after Bonferroni multiple testing correction with n = 91. Boxes comparing overlap of significant PRS-associated genes are darker green for higher numbers of shared genes between two traits. For quantification, see **Supplementary Table 3**.

We further compared PRS-associated genes across traits by calculating the Pearson’s correlation coefficient of the effect size of PRS association for any two traits within each T cell subtype. As expected, genes associated with the PRSs for lymphocyte and white blood cell counts were highly correlated in all four T cell subtypes (see **Figure 2**, quantification in **Supplementary Table 3**). Within a particular trait category (immune function, autoimmune diseases, and neurological disorders), significant correlations between trait-associated gene expression patterns were generally positive. Trends in correlation coefficients also remained the same across T cell subtypes, with few exceptions. However, the gene expression signature associated with the PRS for stroke was negatively correlated with other neurological conditions such as Parkinson’s disease, amyotrophic lateral sclerosis, and epilepsy.

### 3.3 Pathway analysis of PRS-associated genes

**Figure 3:**
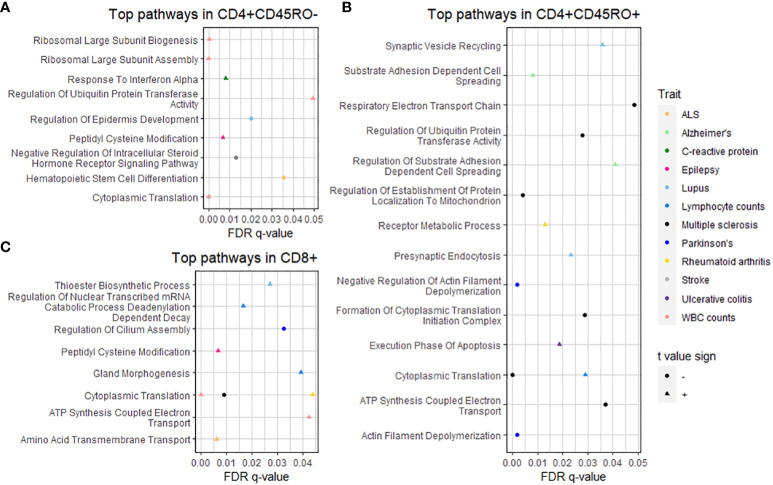
GSEA of PRS-associated genes by T cell subtype. Dot plots show pathways significantly over- or under-represented after multiple testing correction for **(A)** CD4+CD45RO-, **(B)** CD4+CD45RO+, and **(C)** CD8+ subtypes. Trait is shown by color (see legend at right), q-value after multiple testing correction is shown by position on the x-axis, and shape denotes whether the t-value sign for the pathway is negative (circle, meaning the pathway is under-represented) or positive (triangle, meaning the pathway is over-represented). Statistics of significant GSEA pathways are found in **Supplementary Table 4**.

To interrogate the functional connections of PRS-associated genes, we used Gene Set Enrichment Analysis (22) (GSEA) to detect biological pathways in the GO Biological Processes database (23) over-represented or under-represented by genes from our dataset. Input genes for GSEA were ranked by the *t* value for association with the PRS. Pathways with a false discovery rate q-value under 0.05 are shown in the dot plots in **Figure 3** organized by cell type and colored by trait (significant GSEA pathways can also be viewed in **Supplementary Table 4**). Interestingly, some of these pathways relate to functions without a strongly established biological connection to disease pathology. For example, the “presynaptic endocytosis” and “synaptic vesicle recycling” pathways, which we would expect to be potentially dysregulated in some neurological disorders, are significant among genes positively associated with the PRS for systemic lupus erythematosus.

Closer interrogation of several pathways revealed genes involved in T cell and other immune cell functions. For “substrate adhesion-dependent cell spreading”, associated with the PRS for AD in CD4+CD45RO+, genes included C1QBP and ITGA4. The “hematopoietic stem cell differentiation” pathway, associated with the PRS for ALS in CD4+CD45RO- T cells, includes pro-inflammatory interleukin genes IL1A, IL1B, and IL6, and the CXCR4 chemokine receptor that may spur T cell migration into CNS tissue, especially during neurodegenerative disease (3). SELL, a gene that allows naïve T cells to exit the bloodstream into peripheral lymph nodes (50), is part of the “response to interferon alpha” pathway associated with the PRS for C-reactive protein levels in CD4+CD45RO- (presumably naïve) T cells in our data.

These findings better elucidate T cell functional changes for disorders whose relation to T cell biology is less understood, such as AD or epilepsy. Pathway analysis also sheds light on potential T cell mechanisms for traits whose connection to adaptive immunity is well known, such as lymphocyte counts or C-reactive protein levels. Finally, these data imply that the extent and nature of T cell activity in disease pathology may depend in part on individual polygenic risk for these conditions.

### 3.4 Comparing abundance of PRS-associated genes between CD4+ and CD8+ T cell subtypes

Some conditions feature pathological mechanisms that are unique to a particular T cell subtype or favored by one T cell subtype over another. Many autoimmune disease traits, for example, are driven more by CD4+ T cell-mediated pathology than CD8+ (51), while recent single-cell sequencing research in neurodegenerative disease patients has highlighted mechanisms of inflammation and cytotoxicity in CD8+ T cell clusters (2, 3, 52). We sought to determine whether similar patterns existed in our genotype-phenotype correlation studies, based on the abundance of PRS-associated genes in CD4+ and CD8+ T cell subtypes by trait.

**Figure 4:**
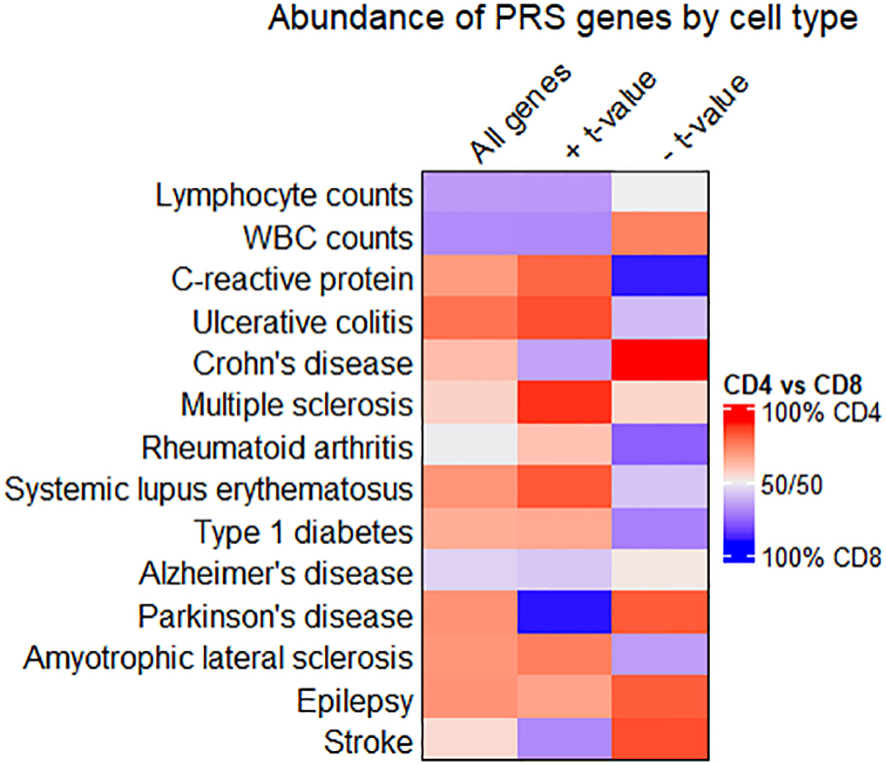
Comparing enrichment of PRS-associated genes between CD4+ and CD8+ T cell subtypes. Heatmap showing the relative abundance of all PRS-associated genes (left column), genes with a positive *t*-value (middle column), or genes with a negative t-value (right column) in CD4+ or CD8+ T cell subtypes. Cells are colored red when most genes are in CD4+ T cell subtypes, and blue when most genes are in CD8+. Quantities associated with this heatmap are available in **Supplementary Table 5**.

**Figure 4** shows that autoimmune disease traits (ulcerative colitis through type 1 diabetes) have most PRS-associated genes in CD4+ T cell subtypes, pairing well with previous observations of CD4+ T cell involvement in these conditions (51). However, when only considering genes inversely related to the PRS (denoted by a negative t-value and shown in the rightmost column), several traits have the majority of these genes in CD8+ T cell subtypes. For individuals with high polygenic risk for autoimmune disease, this suggests a shift towards a downregulated gene expression pattern in CD8+ T cells, even as CD4+ T cells ramp up expression of many genes. Lymphocyte and white blood cell count traits have a higher percentage of all PRS-associated genes in CD8+ T cell subtypes. For several traits, the relative percentage of genes in CD4+ versus CD8+ T cell subtypes differs widely by the *t*-value sign (for quantification, see **Supplementary Table 5**).

We looked further into PRS-associated genes on the extreme ends of the t value distribution for traits where gene enrichment in CD4+ *vs*. CD8+ T cells differed by *t* value sign. Genes in CD4+ T cells with a negative *t* value for association with the PRS included HAVCR1 in Crohn’s disease. CD4+ genes inversely associated with the PRS for Parkinson’s included DOCK8 and CD59. Among CD8+ genes that scaled proportionally with the PRS for Parkinson’s disease, top genes included GZMM and IL2RB. Genes related to the PRS for C-reactive protein levels include IL16, IL10RA, CASP1, and PPM1A. PPM1A, while upregulated in CD4+ T cells of individuals with high PRS for C-reactive protein levels, is downregulated in CD8+ T cells for the same individuals in our data.

## 4 Discussion

Our experiments show that T cell gene expression phenotypes can change with respect to polygenic risk for disease. We calculated a PRS for AD in our cohort and estimated its predictive value for disease, then calculated PRSs for 13 other diseases and traits. Correlating PRSs with gene expression, and determining PRS-associated genes with high overlap across traits and T cell subtypes, detected genes involved in key aspects of T cell signaling and activation. We explored how PRS-associated T cell transcriptomic signatures compared between traits, and biological pathways and processes represented by PRS-associated genes. Finally, we found that several traits displayed differential polarization of their PRS-associated genes towards CD4+ or CD8+ T cell subtypes, even when considering the direction of association between the PRS and gene expression. While several groups have previously detailed changes in T cell gene expression related to individual genetic variants, our analyses here show that many disease-associated variants can have an aggregate effect on T cell transcriptomic phenotypes as well.

The predictive value of the AD PRS in our cohort for disease status provided helpful confirmatory results in light of other PRS studies. Just as we detected associations between the AD PRS and measures of AD pathology, polygenic risk for AD has been shown elsewhere to correlate with neuropathological phenotypes such as higher amyloid burden as measured with positron emission tomography (PET) (53, 54), volume loss in brain regions such as the hippocampus and entorhinal cortex as seen on MRI (55, 56), and levels of phosphorylated tau or amyloid beta in plasma or cerebrospinal fluid (57–59). Less understood is the way immune cell behavior is shaped by disease-associated genetic variants in aggregate. While our dataset derives from a primarily AD participant cohort, T cell gene expression changes were observed in association with the PRS for eighteen other traits. Just as other studies have shown PRS-associated changes in cognitive performance at ages far below the typical onset of AD (53, 56), our gene expression findings suggest that T cell transcriptomic identity could be altered by a genetic landscape predisposing to conditions like lupus or epilepsy, even without clinical signs.

Correlating transcriptomic phenotypes across traits in our dataset generally showed that traits within a particular category, such as autoimmune disease, had positively correlated gene expression patterns. Some exceptions exist, as for stroke, whose PRS-associated gene expression profile correlated poorly with other neurological conditions. Dissecting the relationship between PRSs across traits, or PRS-associated phenotypes, could be informative in other cohorts. We know that the overall genetic architecture of several autoimmune disease traits features a notable degree of overlap (60), as do several subtypes of dementia (61). Our study suggests that PRS-associated T cell genes may reveal similar polygenic risk-mediated phenotypes across several related disease traits. For autoimmune disease traits, this overlap could reflect common T cell autoreactivity mechanisms regardless of the antigenic target, such as upregulation of genes related to inflammation or proliferation. If this is the case, therapeutic strategies targeting PRS-associated T cell genes could show efficacy in a range of autoimmune conditions. Such an approach could also be effective for neurological diseases, where immune-targeted therapies that reduce pathology in one disease context, such as AD, could be repurposed for ALS or epilepsy.

Our pathway analysis results hinted at the importance of profiling genotype-phenotype correlations in disease-relevant tissue. Several functionally intriguing pathways included genes known to affect migration and homing in T cells. In several disease states, T cell gene expression and clonality can change dramatically upon entry of T cells into target tissue. For example, in Parkinson’s and Lewy body dementia patients, T cells isolated from CSF presented a distinct transcriptomic signature from peripheral blood T cells, including upregulation of the chemokine receptor CXCR4 (3). Often, genetically-regulated gene expression changes in T cells are specific to particular T cell subtypes (6, 8–11, 62) or activation states *in vitro* (8, 9, 63), suggesting that genotype-phenotype correlations specific to tissue microenvironment may exist as well. Our use of peripheral T cells in an AD patient cohort, as opposed to CNS-derived T cells, represents a limitation to the generalizability of our findings in a neurodegenerative disease context.

The use of many contributing SNPs as input to the PRS often results in more generalized findings, compared to the single SNP-single gene approach of eQTL studies. This was certainly the case in our results, as only two PRS-associated genes remained significant after Bonferroni multiple testing correction at q = 0.05. The GWAS base data used for PRS calculation also generally does not account for the reality of disease subtypes or phenotypic variability between patients, which is especially prevalent in autoimmune diseases. While some diseases show a similar landscape of genetic risk across phenotypic subtypes (34), future GWAS studies should examine these subgroups more closely, to identify individual variants or trends in polygenic risk associated with specific manifestations of disease progression. Our low sample size in comparison to most genotype-phenotype correlation studies likely limits well-powered detection of PRS-associated genes after multiple testing correction. Future studies seeking to robustly detect PRS-associated genes, especially in multiple tissues or cell types, should likely have a sample size of several hundred or more. Newer options for reduced cost genotyping and RNA-sequencing should make this approach feasible.

Our use of CD45RO as a marker to differentiate naïve from memory T cells also represents a limitation to the interpretability of our findings, as T cell memory populations that re-express CD45RA (41) would be labeled as naïve by our sorting strategy. These memory T cells subtypes, known as TEMRA, have particular importance in neurodegenerative disease contexts (2). CD4+ T cell populations also have several subtypes distinguished by specific transcription factors and secreted cytokines, including Th1, Th2, Th17, regulatory T, and others. In several diseases we have included here, pathology is mediated far more by some CD4+ T cell subtypes than others, especially for autoimmune diseases. Other conditions involve a deficiency in one or more of these subtypes, such as regulatory T cells in ALS (64). It is likely that our sorting strategy misses some PRS-mediated gene expression changes that would only be seen in specific CD4+ T cell subtypes. Importantly, the critical contribution of B cells to the pathology of several autoimmune diseases is not reflected in our choice of immune cell types, and should be examined. Future studies seeking to identify PRS-associated genes in multiple cell types should be aware of sources of nuance in cell subtype markers, or use methods such as single-cell RNA-sequencing or cellular indexing of transcriptomes and epitopes (CITE-seq) to comprehensively profile markers for robust cell type identification.

In future research, studies that arise from other cohorts involving collection of genomic and gene expression data will be vital tools for comparing eQTL with polygenic risk-mediated transcriptomic phenotypes, especially those that collect gene expression data from specific tissues or cell types. These studies should be sufficiently large to generate well-powered results for PRS calculation and correlation of PRS with other phenotypes. Existing datasets from projects such as GTEx (65), which has already extensively profiled tissue-specific eQTL, could also be mined for polygenic risk-mediated gene expression changes in a variety of disease settings. The number of studies collecting genome sequencing or genotyping data alongside one or more quantitative traits will continue to expand, for AD and other disease cohorts. The utilization of PRSs in genotype-phenotype correlation studies will be invaluable for diseases such as AD, where the contribution of T cells is coming to light.

## Data availability statement

All PRS-associated genes generated in this study are found in **Supplementary Table 1**. The original gene expression count matrix is in **Supplementary Table 6** of this publication, and on Zenodo (DOI: 10.5281/zenodo.10822958). The datasets containing individual-level genomic data, including genetic variants and PRSs, are not readily available because of ethical and privacy restrictions, including restrictions due to participant identifiability. Requests to access the datasets should be directed to Badri Vardarajan, bnv2103@cumc.columbia.edu, or on synapse.org with accession number syn17008936. Code used in this publication can be found at https://github.com/ddressman91/Frontiers_PRS_Tcells.

## Ethics statement

The studies involving humans were approved by IRB-AAAS9463 Columbia University. The studies were conducted in accordance with the local legislation and institutional requirements. The participants provided their written informed consent to participate in this study.

## Author contributions

DD: Writing – review & editing, Writing – original draft, Visualization, Methodology, Formal Analysis. ST: Writing – review & editing, Methodology, Formal Analysis. LY: Writing – review & editing, Methodology, Formal Analysis. JS: Writing – review & editing, Resources, Data curation. DB: Writing – review & editing, Resources, Funding acquisition, Data curation. WE: Writing – review & editing, Writing – original draft, Visualization, Supervision, Methodology, Funding acquisition, Formal Analysis, Conceptualization. BV: Writing – review & editing, Writing – original draft, Visualization, Supervision, Methodology, Formal Analysis, Conceptualization.

## Supplementary material

Supplementary Figure 1 Validation of the AD PRS against diagnostic data and pathological trait measurements. **A)** ROC curves, colored by the SNP p-value threshold used to calculate the PRS, showing the predictive value of each set of PRSs for AD against clinical diagnosis of AD. **B)** ROC curves showing the predictive value of the AD PRS against pathological diagnosis of AD. **C)** Comparison of the PRS score distribution by Braak score. **D-F)** Scatter plot of the AD PRS against quantification of amyloid burden, tau tangle score, and global pathology score (gpath).

Supplementary Figure 2 Visualization of cell clustering and gene expression trends in single-cell RNA-sequencing of peripheral T cells. **A)** UMAP projection of T cell subclusters from the comparison dataset, with clusters colored according to the legend at right. **B-F)** Violin plots comparing gene expression between AD patients and controls for MAPK1 in CD8+ memory, C1QBP in CD4+ memory, DBNL in CD4+ naïve, ITSN2 in CD8+ memory, and NFATC2 in CD4+ memory T cells.

Supplementary Figure 3 Summary of the overlap among PRS-associated genes across traits and T cell subsets. **A)** Histogram showing the number of genes (y axis) found associated with the PRS for a given number of traits (x axis) across T cell subsets. **B)** Stacked bar chart showing the number of genes (y axis) found associated with the PRS for a given number of traits (x axis) within T cell subsets, with cell type given by color.

Supplementary Table 1 All PRS-associated genes, with associated effect sizes, unadjusted *p*-values, and adjusted *p*-values after multiple testing correction by Benjamini-Hochberg false discovery rate or Bonferroni correction. Each tab is for one trait and one T cell subtype (c1 is CD4+CD45RO-, c2 is CD4+CD45RO+, c3 is CD8+CD45RO-, c4 is CD8+CD45RO+).

Supplementary Table 2 Genes differentially expressed between AD subjects and healthy controls in a comparison dataset of peripheral T cells, with one tab for each T cell subtype. Columns labeled pct.1 and pct.2 represent the percentage of cells in AD subjects and the percentage of cells in healthy controls, respectively, expressing a particular gene. Columns for *p*-value (nominal and adjusted) are also given. The column labeled avg_log2FC refers to the average log base 2 fold change between AD patients and healthy controls for a given gene, with positive values denoting upregulation in AD patients.

Supplementary Table 3 Quantities of Pearson’s r values for are shown above the yellow diagonal. NA values indicate correlations that were insignificant after Bonferroni multiple testing correction. Absolute numbers of shared significant PRS-associated genes between any two traits are shown below the yellow diagonal.

Supplementary Table 4 All GSEA pathways from all traits and T cell subtypes that are significantly over- or under-represented among PRS-associated genes after multiple testing correction. SIZE refers to the number of genes in the pathway, t.value.sign refers to the whether the pathway is over-represented (+) or under-represented (-) by genes positively correlated with the PRS for a given trait.

Supplementary Table 5 Quantities used to obtain the heatmap, including absolute counts of PRS-associated genes in CD4+ and CD8+ subtypes for each trait, percentage of all PRS-associated genes for a given trait, and percent differences in PRS-associated gene count between CD4+ and CD8+ subtypes for a given trait.

Supplementary Table 6 Gene expression count matrix with raw RNA expression counts used to detect PRS-associated genes.

## References

[1] Merlini M Kirabali T Kulic L Nitsch RM Ferretti MT. Extravascular CD3+ T cells in brains of alzheimer disease patients correlate with tau but not with amyloid pathology: an immunohistochemical study. Neurodegener Dis. (2018) 18:49–56. doi: 10.1159/000486200 29402847

[2] Gate D Saligrama N Leventhal O Yang AC Unger MS Middeldorp J. Clonally expanded CD8 T cells patrol the cerebrospinal fluid in Alzheimer’s disease. Nature. (2020) 577:399–404. doi: 10.1038/s41586-019-1895-7 31915375 PMC7445078

[3] Gate D Tapp E Leventhal O Shahid M Nonninger TJ Yang AC. CD4+ T cells contribute to neurodegeneration in Lewy body dementia. Science. (2021) 374:868–74. doi: 10.1126/science.abf7266 PMC9122025 34648304

[4] Dressman D Buttrick T Cimpean M Bennett D Menon V Bradshaw EM. Genotype–phenotype correlation of T-cell subtypes reveals senescent and cytotoxic genes in Alzheimer’s disease. Hum Mol Genet. (2022) 31:3355–66. doi: 10.1093/hmg/ddac126 PMC9523563 35640154

[5] Raj T Rothamel K Mostafavi S Ye C Lee MN Replogle JM. Polarization of the effects of autoimmune and neurodegenerative risk alleles in leukocytes. Science. (2014) 344:519–23. doi: 10.1126/science.1249547 PMC4910825 24786080

[6] Kasela S Kisand K Tserel L Kaleviste E Remm A Fischer K. Pathogenic implications for autoimmune mechanisms derived by comparative eQTL analysis of CD4+ versus CD8+ T cells. PloS Genet. (2017) 13:e1006643. doi: 10.1371/journal.pgen.1006643 28248954 PMC5352142

[7] Chen L Ge B Casale FP Vasquez L Kwan T Garrido-Martín D. Genetic drivers of epigenetic and transcriptional variation in human immune cells. Cell. (2016) 167:1398–1414.e24. doi: 10.1016/j.cell.2016.10.026 27863251 PMC5119954

[8] Schmiedel BJ Singh D Madrigal A Valdovino-Gonzalez AG White BM Zapardiel-Gonzalo J. Impact of genetic polymorphisms on human immune cell gene expression. Cell. (2018) 175:1701–1715.e16. doi: 10.1016/j.cell.2018.10.022 30449622 PMC6289654

[9] Schmiedel BJ Gonzalez-Colin C Fajardo V Rocha J Madrigal A Ramírez-Suástegui C. Single-cell eQTL analysis of activated T cell subsets reveals activation and cell type–dependent effects of disease-risk variants. Sci Immunol. (2022) 7:eabm2508. doi: 10.1126/sciimmunol.abm2508 35213211 PMC9035271

[10] Nathan A Asgari S Ishigaki K Valencia C Amariuta T Luo Y. Single-cell eQTL models reveal dynamic T cell state dependence of disease loci. Nature. (2022) 606:120–8. doi: 10.1038/s41586-022-04713-1 PMC9842455 35545678

[11] Soskic B Cano-Gamez E Smyth DJ Ambridge K Ke Z Matte JC. Immune disease risk variants regulate gene expression dynamics during CD4+ T cell activation. Nat Genet. (2022) 54:817–26. doi: 10.1038/s41588-022-01066-3 PMC9197762 35618845

[12] Fromer M Roussos P Sieberts SK Johnson JS Kavanagh DH Perumal TM. Gene expression elucidates functional impact of polygenic risk for schizophrenia. Nat Neurosci. (2016) 19:1442–53. doi: 10.1038/nn.4399 PMC5083142 27668389

[13] Tasaki S Gaiteri C Mostafavi S De Jager PL Bennett DA. The molecular and neuropathological consequences of genetic risk for alzheimer’s dementia. Front Neurosci. (2018) 12:699. doi: 10.3389/fnins.2018.00699 30349450 PMC6187226

[14] Crawford K Leonenko G Baker E Grozeva D Lan-Leung B Holmans P. Golgi apparatus, endoplasmic reticulum and mitochondrial function implicated in Alzheimer’s disease through polygenic risk and RNA sequencing. Mol Psychiatry. (2023) 28:1327–36. doi: 10.1038/s41380-022-01926-8 PMC10005937 36577842

[15] Bennett DA Buchman AS Boyle PA Barnes LL Wilson RS Schneider JA. Religious orders study and rush memory and aging project. J Alzheimers Dis JAD. (2018) 64:S161–89. doi: 10.3233/JAD-179939 PMC6380522 29865057

[16] Soumillon M Cacchiarelli D Semrau S van Oudenaarden A Mikkelsen TS. Characterization of directed differentiation by high-throughput single-cell RNA-Seq. bioRxiv. (2014), 003236. doi: 10.1101/003236v1

[17] Robinson MD McCarthy DJ Smyth GK. edgeR: a Bioconductor package for differential expression analysis of digital gene expression data. Bioinformatics. (2010) 26:139–40. doi: 10.1093/bioinformatics/btp616 PMC2796818 19910308

[18] Law CW Chen Y Shi W Smyth GK. voom: precision weights unlock linear model analysis tools for RNA-seq read counts. Genome Biol. (2014) 15:R29. doi: 10.1186/gb-2014-15-2-r29 24485249 PMC4053721

[19] Purcell S Neale B Todd-Brown K Thomas L Ferreira MAR Bender D. PLINK: A tool set for whole-genome association and population-based linkage analyses. Am J Hum Genet. (2007) 81:559–75. doi: 10.1086/519795 PMC1950838 17701901

[20] Choi SW O’Reilly PF. PRSice-2: Polygenic Risk Score software for biobank-scale data. GigaScience. (2019) 8:giz082. doi: 10.1093/gigascience/giz082 31307061 PMC6629542

[21] Robin X Turck N Hainard A Tiberti N Lisacek F Sanchez JC. pROC: an open-source package for R and S+ to analyze and compare ROC curves. BMC Bioinf. (2011) 12:1–8. doi: 10.1186/1471-2105-12-77 PMC3068975 21414208

[22] Subramanian A Tamayo P Mootha VK Mukherjee S Ebert BL Gillette MA. Gene set enrichment analysis: A knowledge-based approach for interpreting genome-wide expression profiles. Proc Natl Acad Sci. (2005) 102:15545–50. doi: 10.1073/pnas.0506580102 PMC1239896 16199517

[23] Ashburner M Ball CA Blake JA Botstein D Butler H Cherry JM. Gene Ontology: tool for the unification of biology. Nat Genet. (2000) 25:25–9. doi: 10.1038/75556 PMC3037419 10802651

[24] Gu Z. Complex heatmap visualization. iMeta. (2022) 1:e43. doi: 10.1002/imt2.43 PMC10989952 38868715

[25] Wickham H. ggplot2. WIREs Comput Stat. (2011) 3:180–5. doi: 10.1002/wics.147

[26] Xu H Jia J. Single-cell RNA sequencing of peripheral blood reveals immune cell signatures in alzheimer’s disease. Front Immunol. (2021) 12:645666. doi: 10.3389/fimmu.2021.645666 34447367 PMC8382575

[27] Satija R Farrell JA Gennert D Schier AF Regev A. Spatial reconstruction of single-cell gene expression data. Nat Biotechnol. (2015) 33:495–502. doi: 10.1038/nbt.3192 25867923 PMC4430369

[28] Kunkle BW Grenier-Boley B Sims R Bis JC Damotte V Naj AC. Genetic meta-analysis of diagnosed Alzheimer’s disease identifies new risk loci and implicates Aβ, tau, immunity and lipid processing. Nat Genet. (2019) 51:414–30. doi: 10.1038/s41588-019-0358-2 PMC6463297 30820047

[29] Ravizza T Gagliardi B Noé F Boer K Aronica E Vezzani A. Innate and adaptive immunity during epileptogenesis and spontaneous seizures: Evidence from experimental models and human temporal lobe epilepsy. Neurobiol Dis. (2008) 29:142–60. doi: 10.1016/j.nbd.2007.08.012 17931873

[30] Yang C Hawkins KE Doré S Candelario-Jalil E. Neuroinflammatory mechanisms of blood-brain barrier damage in ischemic stroke. Am J Physiol-Cell Physiol. (2019) 316:C135–53. doi: 10.1152/ajpcell.00136.2018 PMC6397344 30379577

[31] Sakaue S Kanai M Tanigawa Y Karjalainen J Kurki M Koshiba S. A cross-population atlas of genetic associations for 220 human phenotypes. Nat Genet. (2021) 53:1415–24. doi: 10.1038/s41588-021-00931-x PMC12208603 34594039

[32] de Lange KM Moutsianas L Lee JC Lamb CA Luo Y Kennedy NA. Genome-wide association study implicates immune activation of multiple integrin genes in inflammatory bowel disease. Nat Genet. (2017) 49:256–61. doi: 10.1038/ng.3760 PMC5289481 28067908

[33] International Multiple Sclerosis Genetics Consortium (IMSGC) Beecham AH Patsopoulos NA Xifara DK Davis MF Kemppinen A. Analysis of immune-related loci identifies 48 new susceptibility variants for multiple sclerosis. Nat Genet. (2013) 45:1353–60. doi: 10.1038/ng.2770 PMC3832895 24076602

[34] Ishigaki K Sakaue S Terao C Luo Y Sonehara K Yamaguchi K. Multi-ancestry genome-wide association analyses identify novel genetic mechanisms in rheumatoid arthritis. Nat Genet. (2022) 54:1640–51. doi: 10.1038/s41588-022-01213-w PMC10165422 36333501

[35] Langefeld CD Ainsworth HC Graham DSC Kelly JA Comeau ME Marion MC. Transancestral mapping and genetic load in systemic lupus erythematosus. Nat Commun. (2017) 8:16021. doi: 10.1038/ncomms16021 28714469 PMC5520018

[36] Robertson CC Inshaw JRJ Onengut-Gumuscu S Chen WM Cruz DFS Yang H. Fine-mapping, trans-ancestral, and genomic analyses identify causal variants, cells, genes, and drug targets for type 1 diabetes. Nat Genet. (2021) 53:962–71. doi: 10.1038/s41588-021-00880-5 PMC8273124 34127860

[37] Chang D Nalls MA Hallgrímsdóttir IB Hunkapiller J van der Brug M Cai F. A meta-analysis of genome-wide association studies identifies 17 new Parkinson’s disease risk loci. Nat Genet. (2017) 49:1511–6. doi: 10.1038/ng.3955 PMC5812477 28892059

[38] van Rheenen W van der Spek RAA Bakker MK van Vugt JJFA Hop PJ Zwamborn RAJ. Common and rare variant association analyses in amyotrophic lateral sclerosis identify 15 risk loci with distinct genetic architectures and neuron-specific biology. Nat Genet. (2021) 53:1636–48. doi: 10.1038/s41588-021-00973-1 PMC8648564 34873335

[39] Abou-Khalil B Auce P Avbersek A Bahlo M Balding DJ Bast T. Genome-wide mega-analysis identifies 16 loci and highlights diverse biological mechanisms in the common epilepsies. Nat Commun. (2018) 9:5269. doi: 10.1038/s41467-018-07524-z PMC6288131 30531953

[40] Malik R Chauhan G Traylor M Sargurupremraj M Okada Y Mishra A. Multiancestry genome-wide association study of 520,000 subjects identifies 32 loci associated with stroke and stroke subtypes. Nat Genet. (2018) 50:524–37. doi: 10.1038/s41588-018-0058-3 PMC5968830 29531354

[41] Tian Y Babor M Lane J Schulten V Patil VS Seumois G. Unique phenotypes and clonal expansions of human CD4 effector memory T cells re-expressing CD45RA. Nat Commun. (2017) 8:1473. doi: 10.1038/s41467-017-01728-5 29133794 PMC5684192

[42] Hu S Xie Z Onishi A Yu X Jiang L Lin J. Profiling the human protein-DNA interactome reveals MAPK1 as a transcriptional repressor of interferon signalling. Cell. (2009) 139:610–22. doi: 10.1016/j.cell.2009.08.037 PMC2774939 19879846

[43] Tian H Wang G Wang Q Zhang B Jiang G Li H. Complement C1q binding protein regulates T cells’ mitochondrial fitness to affect their survival, proliferation, and anti–tumor immune function. Cancer Sci. (2022) 113:875–90. doi: 10.1111/cas.15261 PMC8898709 34978120

[44] Rocha-Perugini V Gordon-Alonso M Sánchez-Madrid F. Role of drebrin at the immunological synapse. Adv Exp Med Biol. (2017) 1006:271–80. doi: 10.1007/978-4-431-56550-5_15 PMC6485630 28865025

[45] Huang W Lin W Chen B Zhang J Gao P Fan Y. NFAT and NF-κB dynamically co-regulate TCR and CAR signaling responses in human T cells. Cell Rep. (2023) 42. doi: 10.1016/j.celrep.2023.112663 37347664

[46] Locard-Paulet M Voisinne G Froment C Goncalves Menoita M Ounoughene Y Girard L. LymphoAtlas: a dynamic and integrated phosphoproteomic resource of TCR signaling in primary T cells reveals ITSN2 as a regulator of effector functions. Mol Syst Biol. (2020) 16:e9524. doi: 10.15252/msb.20209524 32618424 PMC7333348

[47] McCandless EE Zhang B Diamond MS Klein RS. CXCR4 antagonism increases T cell trafficking in the central nervous system and improves survival from West Nile virus encephalitis. Proc Natl Acad Sci U S A. (2008) 105:11270–5. doi: 10.1073/pnas.0800898105 PMC2495012 18678898

[48] Heng AHS Han CW Abbott C McColl SR Comerford I. Chemokine-driven migration of pro-inflammatory CD4+ T cells in CNS autoimmune disease. Front Immunol. (2022) 13:817473. doi: 10.3389/fimmu.2022.817473 35250997 PMC8889115

[49] Bunton-Stasyshyn RKA Saccon RA Fratta P Fisher EMC. SOD1 function and its implications for amyotrophic lateral sclerosis pathology: new and renascent themes. Neuroscientist. (2015) 21:519–29. doi: 10.1177/1073858414561795 25492944

[50] Watson HA Durairaj RRP Ohme J Alatsatianos M Almutairi H Mohammed RN. L-selectin enhanced T cells improve the efficacy of cancer immunotherapy. Front Immunol. (2019) 10:1321. doi: 10.3389/fimmu.2019.01321 31249570 PMC6582763

[51] Deng Q Luo Y Chang C Wu H Ding Y Xiao R. The emerging epigenetic role of CD8+T cells in autoimmune diseases: A systematic review. Front Immunol. (2019) 10:856. doi: 10.3389/fimmu.2019.00856 31057561 PMC6482221

[52] Olah M Menon V Habib N Taga MF Ma Y Yung CJ. Single cell RNA sequencing of human microglia uncovers a subset associated with Alzheimer’s disease. Nat Commun. (2020) 11:6129. doi: 10.1038/s41467-020-19737-2 33257666 PMC7704703

[53] Mormino EC Sperling RA Holmes AJ Buckner RL Jager PLD Smoller JW. Polygenic risk of Alzheimer disease is associated with early- and late-life processes. Neurology. (2016) 87:481–8. doi: 10.1212/WNL.0000000000002922 PMC4970660 27385740

[54] Tan CH Bonham LW Fan CC Mormino EC Sugrue LP Broce IJ. Polygenic hazard score, amyloid deposition and Alzheimer’s neurodegeneration. Brain. (2019) 142:460–70. doi: 10.1093/brain/awy327 PMC6351776 30689776

[55] Desikan RS Fan CC Wang Y Schork AJ Cabral HJ Cupples LA. Genetic assessment of age-associated Alzheimer disease risk: Development and validation of a polygenic hazard score. PloS Med. (2017) 14:e1002258. doi: 10.1371/journal.pmed.1002289 28323831 PMC5360219

[56] Axelrud LK Sato JR Santoro ML Talarico F Pine DS Rohde LA. Genetic risk for Alzheimer’s disease and functional brain connectivity in children and adolescents. Neurobiol Aging. (2019) 82:10–7. doi: 10.1016/j.neurobiolaging.2019.06.011 PMC7658444 31376729

[57] Martiskainen H Helisalmi S Viswanathan J Kurki M Hall A Herukka SK. Effects of Alzheimer’s disease-associated risk loci on cerebrospinal fluid biomarkers and disease progression: a polygenic risk score approach. J Alzheimers Dis JAD. (2015) 43:565–73. doi: 10.3233/JAD-140777 25096612

[58] Cruchaga C Del-Aguila JL Saef B Black K Fernandez MV Budde J. Polygenic risk score of sporadic late-onset Alzheimer’s disease reveals a shared architecture with the familial and early-onset forms. Alzheimers Dement J Alzheimers Assoc. (2018) 14:205–14. doi: 10.1016/j.jalz.2017.08.013 PMC5803427 28943286

[59] Zettergren A Lord J Ashton NJ Benedet AL Karikari TK Lantero Rodriguez J. Association between polygenic risk score of Alzheimer’s disease and plasma phosphorylated tau in individuals from the Alzheimer’s Disease Neuroimaging Initiative. Alzheimers Res Ther. (2021) 13:17. doi: 10.1186/s13195-020-00754-8 33419453 PMC7792087

[60] Richard-Miceli C Criswell LA. Emerging patterns of genetic overlap across autoimmune disorders. Genome Med. (2012) 4:6. doi: 10.1186/gm305 22284131 PMC3334554

[61] Ferrari R Wang Y Vandrovcova J Guelfi S Witeolar A Karch CM. Genetic architecture of sporadic frontotemporal dementia and overlap with Alzheimer’s and Parkinson’s diseases. J Neurol Neurosurg Psychiatry. (2017) 88:152–64. doi: 10.1136/jnnp-2016-314411 PMC5237405 27899424

[62] van der Wijst MGP Brugge H de Vries DH Deelen P Swertz MA Franke L. Single-cell RNA sequencing identifies cell type-specific cis-eQTLs and co-expression QTLs. Nat Genet. (2018) 50:493–7. doi: 10.1038/s41588-018-0089-9 PMC5905669 29610479

[63] Ye CJ Feng T Kwon HK Raj T Wilson M Asinovski N. Intersection of population variation and autoimmunity genetics in human T cell activation. Science. (2014) 345:1254665. doi: 10.1126/science.1254665 25214635 PMC4751028

[64] Beers DR Zhao W Wang J Zhang X Wen S Neal D. ALS patients’ regulatory T lymphocytes are dysfunctional, and correlate with disease progression rate and severity. JCI Insight. (2017) 2(5):e89530. doi: 10.1172/jci.insight.89530 PMC5333967 28289705

[65] Aguet F Brown AA Castel SE Davis JR He Y Jo B. Genetic effects on gene expression across human tissues. Nature. (2017) 550:204–13. doi: 10.1038/nature24277 PMC5776756 29022597

