# Supplementary material for: Polygenic risk associated with Alzheimer’s disease and other traits influences genes involved in T cell signaling and activation
Source: Front Immunol. 2024 Mar 25;15:1337831. doi: 10.3389/fimmu.2024.1337831 (PMC10999606; doi:10.3389/fimmu.2024.1337831)
Supplement: Supplementary Figure 1 — Validation of the AD PRS against diagnostic data and pathological trait measurements. A) ROC curves, colored by the SNP p-value threshold used to calculate the PRS, showing the predictive value of each set of PRSs for AD against clinical diagnosis of AD. B) ROC curves showing the predictive value of the AD PRS against pathological diagnosis of AD. C) Comparison of the PRS score distribution by Braak score. D-F) Scatter plot of the AD PRS against quantification of amyloid burden, tau tangle score, and global pathology score (gpath). [file DataSheet_1.docx]

Supplementary Material

# Supplementary Figures


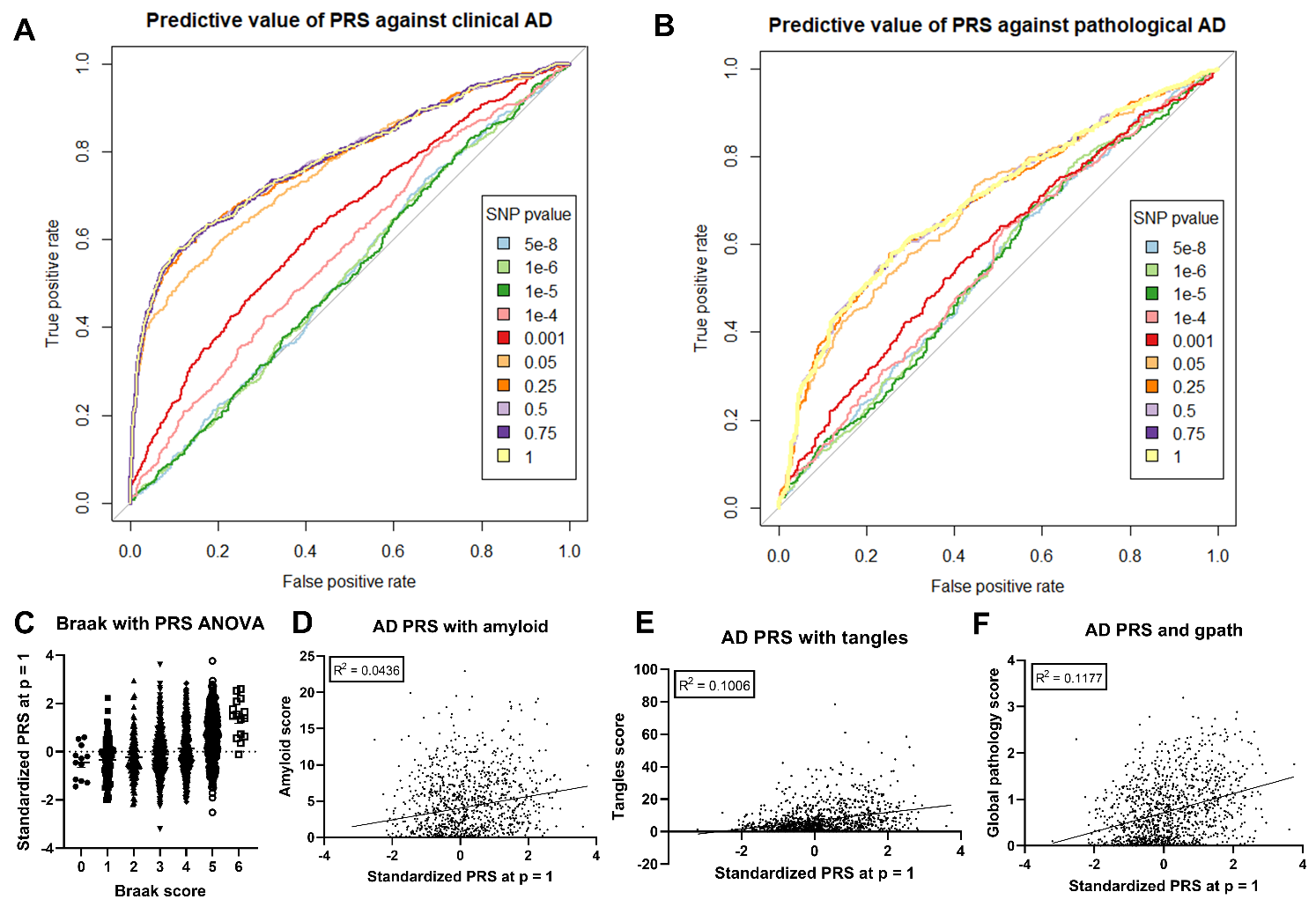


**Supplementary Figure 1:** **Validation of the AD PRS against diagnostic data and pathological trait measurements**. **A)** ROC curves, colored by the SNP p-value threshold used to calculate the PRS, showing the predictive value of each set of PRSs for AD against clinical diagnosis of AD. **B)** ROC curves showing the predictive value of the AD PRS against pathological diagnosis of AD. **C)** Comparison of the PRS score distribution by Braak score. **D-F)** Scatter plot of the AD PRS against quantification of amyloid burden, tau tangle score, and global pathology score (gpath).


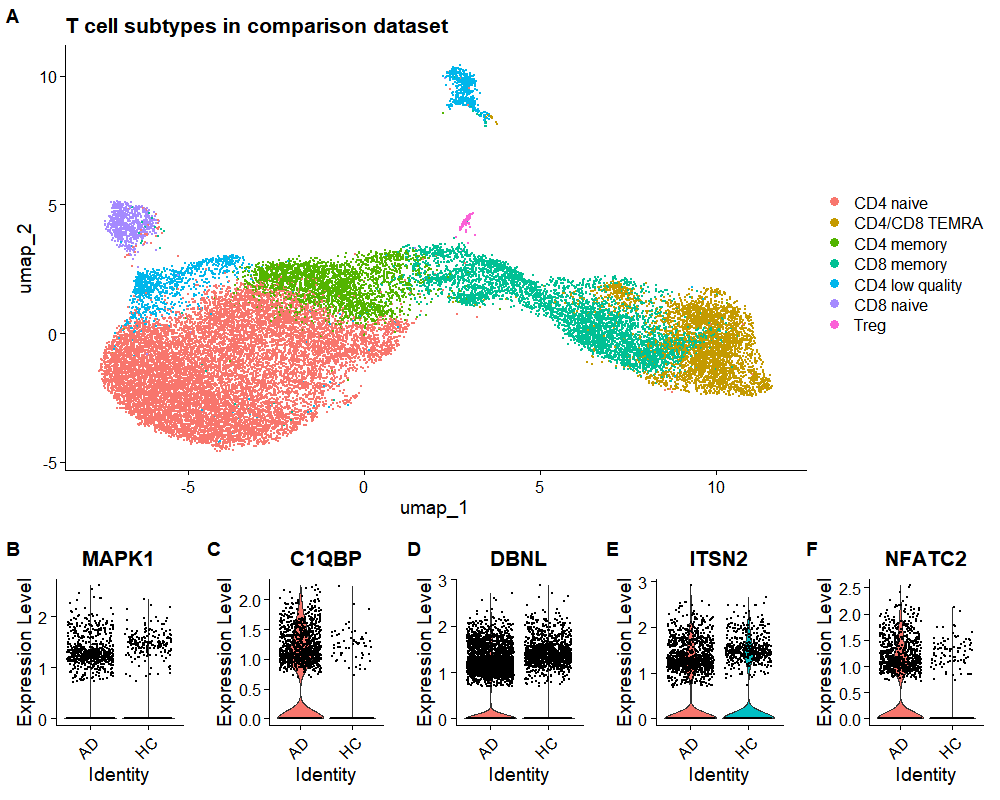


**Supplementary Figure 2: Visualization of cell clustering and gene expression trends in single-cell RNA-sequencing of peripheral T cells. A)** UMAP projection of T cell subclusters from the comparison dataset, with clusters colored according to the legend at right. **B-F)** Violin plots comparing gene expression between AD patients and controls for MAPK1 in CD8+ memory, C1QBP in CD4+ memory, DBNL in CD4+ naïve, ITSN2 in CD8+ memory, and NFATC2 in CD4+ memory T cells.


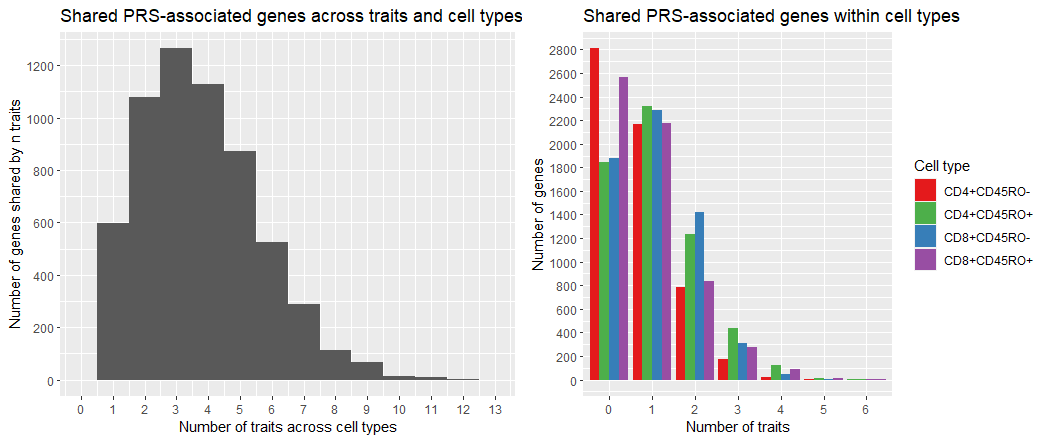


**Supplementary Figure 3: Summary of the overlap among PRS-associated genes across traits and T cell subsets. A)** Histogram showing the number of genes (y axis) found associated with the PRS for a given number of traits (x axis) across T cell subsets. **B)** Stacked bar chart showing the number of genes (y axis) found associated with the PRS for a given number of traits (x axis) within T cell subsets, with cell type given by color.
